# Supplementary material for: Bayesian refinement of protein structures and ensembles against SAXS data using molecular dynamics
Source: PLoS Comput Biol. 2017 Oct 18;13(10):e1005800. doi: 10.1371/journal.pcbi.1005800 (PMC5662244; doi:10.1371/journal.pcbi.1005800)
Supplement: S1 Archive — (BZ2) [file pcbi.1005800.s011.tar.bz2 › gromacs_4.62_bayesian_saxs_refi/share/html/online/g_covar.html]

g\_covar


| g\_covar Main Table of Contents |

**VERSION 4.6  
Sat 19 Jan 2013**

---

### Description

g\_covar calculates and diagonalizes the (mass-weighted)
covariance matrix.
All structures are fitted to the structure in the structure file.
When this is not a run input file periodicity will not be taken into
account. When the fit and analysis groups are identical and the analysis
is non mass-weighted, the fit will also be non mass-weighted.

The eigenvectors are written to a trajectory file (-v).
When the same atoms are used for the fit and the covariance analysis,
the reference structure for the fit is written first with t=-1.
The average (or reference when -ref is used) structure is
written with t=0, the eigenvectors
are written as frames with the eigenvector number as timestamp.

The eigenvectors can be analyzed with g\_anaeig.

Option -ascii writes the whole covariance matrix to
an ASCII file. The order of the elements is: x1x1, x1y1, x1z1, x1x2, ...

Option -xpm writes the whole covariance matrix to an .xpm file.

Option -xpma writes the atomic covariance matrix to an .xpm file,
i.e. for each atom pair the sum of the xx, yy and zz covariances is
written.

Note that the diagonalization of a matrix requires memory and time
that will increase at least as fast as than the square of the number
of atoms involved. It is easy to run out of memory, in which
case this tool will probably exit with a 'Segmentation fault'. You
should consider carefully whether a reduced set of atoms will meet
your needs for lower costs.

### Files

| option | filename | type | description |
| --- | --- | --- | --- |
| **-f** | traj.xtc | Input | Trajectory: xtc trr trj gro g96 pdb cpt |
| **-s** | topol.tpr | Input | Structure+mass(db): tpr tpb tpa gro g96 pdb |
| **-n** | index.ndx | Input, Opt. | Index file |
| **-o** | eigenval.xvg | Output | xvgr/xmgr file |
| **-v** | eigenvec.trr | Output | Full precision trajectory: trr trj cpt |
| **-av** | average.pdb | Output | Structure file: gro g96 pdb etc. |
| **-l** | covar.log | Output | Log file |
| **-ascii** | covar.dat | Output, Opt. | Generic data file |
| **-xpm** | covar.xpm | Output, Opt. | X PixMap compatible matrix file |
| **-xpma** | covara.xpm | Output, Opt. | X PixMap compatible matrix file |

### Other options

| option | type | default | description |
| --- | --- | --- | --- |
| **-[no]h** | bool | no | Print help info and quit || **-[no]version** | bool | no | Print version info and quit || **-nice** | int | 19 | Set the nicelevel || **-b** | time | 0 | First frame (ps) to read from trajectory || **-e** | time | 0 | Last frame (ps) to read from trajectory || **-dt** | time | 0 | Only use frame when t MOD dt = first time (ps) || **-tu** | enum | ps | Time unit: fs, ps, ns, us, ms or s || **-xvg** | enum | xmgrace | xvg plot formatting: xmgrace, xmgr or none || **-[no]fit** | bool | yes | Fit to a reference structure || **-[no]ref** | bool | no | Use the deviation from the conformation in the structure file instead of from the average || **-[no]mwa** | bool | no | Mass-weighted covariance analysis || **-last** | int | -1 | Last eigenvector to write away (-1 is till the last) || **-[no]pbc** | bool | yes | Apply corrections for periodic boundary conditions |

---

http://www.gromacs.org  
gromacs@gromacs.org
